# Supplementary material for: Revealing the spatiotemporal complexity of the magnitude distribution and b-value during an earthquake sequence
Source: Nat Commun. 2022 Aug 29;13:5087. doi: 10.1038/s41467-022-32755-6 (PMC9424211; doi:10.1038/s41467-022-32755-6)
Supplement: Supplementary file 1 — Supplementary Information [file 41467_2022_32755_MOESM1_ESM.pdf]

Supplementary Information for

"Revealing the spatiotemporal complexity of the magnitude distribution and b-value during an earthquake sequence"

Marcus Herrmann, Ester Piegari, Warner Marzocchi

Accompanying supporting datasets (file uploaded separately)

1. Extracted events from the earthquake catalog of Tan et al. [1] with associated cluster IDs obtained in this study.

Contents

Supplementary Notes 2
1 Cluster size statistics . . . 2
2 Additional aspects of MFD and b-value analysis . . . 2
2.1 Sample size and magnitude range . . . 2
2.2 Exponentiality, Lilliefors test, and b-value stability . . . 3
2.3 Influence of magnitude scale . . . 3
2.4 Short-term aftershock incompleteness (STAI) . . . 4
2.5 Dependence on the used catalog . . . 4
2.6 Possible contamination by quarry blasts and explosions . . . 5
Supplementary Figures 6
Supplementary References 19

List of Supplementary Figures

1 Hypocenter density . . . 6
2 Cluster size statistics . . . 7
3 Magnitude statistics of individual clusters using M_L . . . 8
4 Magnitude statistics in individual periods of Cluster 1–3 using M_L . . . 9
5 Reordering Supplementary Fig. 4 temporally by periods . . . 10
6 Temporal evolution of seismicity highlighting short-term aftershock incompleteness (STAI) . 11
7 Influence of STAI on magnitude statistics ('early' and 'mid' period) . . . 12
8 Influence of STAI on magnitude statistics ('late' period) . . . 13
9 Comparison of magnitude statistics for available catalogs that provide M_w . . . 14
10 Spatial distribution of selected subclusters . . . 15
11 Spatial distribution of quarry blasts and CI2016 seismicity . . . 16
12 Histogram of the diurnal occurrence of quarry blasts in the region . . . 17
13 Magnitude statistics in individual periods using only nighttime seismicity . . . 18

Supplementary Notes

Note 1: Cluster size statistics

Supplementary Fig. 2 summarizes the cluster statistics in terms of size and relative proportions for each period, not accounting for short-term aftershock incompleteness (STAI, Note 2.4).

From absolute cluster sizes (Supplementary Fig. 2a), it is evident that

- the 'Norcia' is the most active period;
- only two or three clusters are simultaneously active during the 'Amatrice', 'Visso', and 'Norcia' periods
- all clusters are about equally active in the 'Campotosto' period;

The temporal distribution of each cluster (Supplementary Fig. 2b) reveals that:

- after the Norcia event, all clusters experience a "boost" in seismicity; most of the earthquakes inside and outside the clusters belong to this period, except for C4 and C5;
- C4 and C5 are most active in the Campotosto period.

The relative cluster size (Supplementary Fig. 2c) shows that:

- in each period during the sequence, $\gtrsim 50\%$ of the earthquakes belong to a cluster;
- C1 is the only cluster that is constantly active and never quiet.

Note 2: Additional aspects of MFD and b-value analysis

With particular reference to our analysis, we here discuss further aspects that we consider important when performing and interpreting MFD and b-value analyses.

2.1 Sample size and magnitude range

First and foremost, robust b-value estimates with low uncertainty require a sufficient sample size. In fact, the maximum likelihood estimation assumes that the GR relation holds over a wide magnitude range, typically at least three magnitude units[2]. A MFD that spans less than this range biases the b-value estimate due to a correlation with the largest magnitude and, related to it, a truncated or tapered MFD[3]. In our analysis, we are close to this limit but could not reduce the overall cutoff magnitude below $M_{\mathrm{w}}1.5$ because the $M_{\mathrm{c}}^{\mathrm{Lilliefors}}$ over the whole sequence is not below this level, even in the complete periods, i. e., those unaffected by STAI (see Note 2.4). Although we did not model the MFD with a tapered GR distribution, we can exclude the influence of the corner magnitude on b-value variations because we do not observe a dramatically varying maximum magnitude among the clusters or their temporal subsets. MFD

and b-value variations therefore originate from differences or changes in the entire exponential part of the MFD.

2.2 Exponentiality, Lilliefors test, and b-value stability

For a b-value of 1.0, a magnitude range of three units corresponds to a sample size of 1000. For such rather large sample sizes, the Lilliefors test may still underestimate the departure from exponentiality, i. e., not detect those even if a departure exists[3]. Inferring the robustness of the b-value estimate therefore requires a careful analysis of both the MFD exponentiality and the b-value behavior as function of M_c.

$M_c^{\text{Lilliefors}}$ may only relate to a short-lived exponentiality (i. e., no persistent exponentiality with increasing M_c)[4], for instance in the 'pre-Visso' and 'pre-Campotosto' period of C3 (see Figs. 4 and 5): at a low magnitude level, the p-value indicates an exponential behavior ($p \geq 0.1$), but a departure of exponentiality ($p < 0.1$) at a higher magnitude level. Such short-lived exponentiality may either be due to random fluctuations (especially for small sample sizes) or may have real physical or technical causes (e. g., scaling breaks, incompleteness, or magnitude artifacts)[3,4]. In other cases, $M_c^{\text{Lilliefors}}$ indicates an exponential behavior, but the b-value continuously increases with M_c (e. g., for the 'mid' period, see Supplementary Fig. 7)—this behavior is an indication for STAI (see Note 2.4). For this reason, an inspection of the b-value stability with M_c is always advised.

2.3 Influence of magnitude scale

For a consistent analysis of the MFD, moment magnitudes, M_w, are preferred over local magnitudes, M_L, as discussed in Herrmann and Marzocchi [4].

Supplementary Fig. 3 shows that using M_L introduces a different MFD behavior for the individual clusters. Compared to M_w-based MFDs (Fig. 3), exponentiality of C1 and C2 is rejected for a wider magnitude range. For C3, the exponentiality is only short-lived below $M_L2.0$. This behavior is explained with a stronger under-representation of the M_L scale[4], likely due its intrinsic scaling break toward low magnitudes[5,6]. The b-value at $M_c^{\text{Lilliefors}}$ differs not only in a relative sense (i.e., among the clusters), but also in an absolute sense (being much lower).

Using M_L for the individual periods, this larger discrepancy is reduced (see Supplementary Figs. 4 and 5) and reproduces our main findings that we obtained for M_w (i. e., that the MFD can vary spatially and temporally, but can also remain constant, and that the b-value increases in C1 prior to the Norcia event). However, the b-value behaves differently as function of M_c, owing to the scale change: For C1, the b-value does not increase as strongly anymore with decreasing M_c; for C2 and C3, the b-value is not constant anymore with M_c, but decreases with decreasing M_c.

M_w are important to guarantee consistent analyses such as to obtain a more robust indication for departure of exponentiality (by excluding departures due to M_L's scaling break). Yet, it remains unclear if the magnitude conversion relation of Grünthal et al. [7] used by Tan et al. [1] for converting M_L into M_w represents a suitable choice; it will eventually saturate toward low magnitudes (i. e., overestimate M_w) owing to its polynomial form. This saturation may explain the apparent over-representation at low M_w, favoring an increasing b-value with decreasing M_c as observed in many subsets and in the overall catalog with STAI periods removed (Supplementary Fig. 9).

The relation of Grünthal et al. [7] may not be ideal, but we wanted to use the simplest solution: taking the original M_w estimates provided with the catalog. Alternatives are the relation used in Lolli et al. [8], i. e., the HORUS catalog, a revision of Gasperini et al. [9], or the bilinear relation of Malagnini and Munafò [10]; but using either of those would increase the entropy of our analysis and inevitably the "influence of expert choices" as stated in the main text—a condition that we want to avoid as much as possible as advocated by our study. In this sense, the original catalog creators did the choice for us. The relation of Grünthal et al. [7] is not unsuitable: it is based on European data, which includes data of Italy (1983–2004). Tan et al. [1] did not use this relation directly, but calibrated the constant (using ~500 earthquakes with M_w estimated from regional waveform fitting), as specified in their supplemental material.

2.4 Short-term aftershock incompleteness (STAI)

It is well known that STAI has a large influence on the perceived b-value change[11,12,13]. The Norcia mainshock has the strongest effect, see Supplementary Fig. 7 ('mid' period): For all clusters, the b-value in the 'mid' period continuously increases with increasing M_c, indicating a gradually curved MFD due to incompleteness. Recall from Fig. 4 that the periods excluding STAI had shown the opposite behavior, highlighting the strong influence of STAI on the MFD shape by reducing the estimated b-value. In C1 and C2, the 'early' period has lower b-values than their 'pre-Visso' period (Supplementary Fig. 7), which purposely excluded the STAI effect of the Amatrice event; in C3, this effect is not evident, indicating that the Amatrice event is too far south to influence the detection of earthquakes in the northern part. The Campotosto events in the 'late' period also cause a STAI effect (see Supplementary Fig. 8), albeit only in C2, which is located closest to the Campotosto events in the southern part; C1 and C3 are too far away to observe the same effect. Given these observations, STAI needs to be carefully addressed, e. g., by disregarding incomplete periods, but only as much as necessary to not reduce the number of usable earthquakes unnecessarily. An alternative is using only positive magnitude differences for estimating the b-value because they are not affected by STAI[13].

2.5 Dependence on the used catalog

In Supplementary Fig. 9, we compared the M_w-based magnitude statistics of Tan et al. [1]'s high-resolution catalog with the HORUS catalog for the CI2016 sequence. Event selection

comprises the spatiotemporal extent of the extracted high-resolution catalog, i. e., from 2016-08-15 until 2017-08-15 within the volume it covers. The comparison was performed for two cases: (i) using all events during the sequence, and (ii) using only events in non-STAI periods that are indicated in Supplementary Fig. 6. The analysis shows that the b-value differs considerably between both catalogs (0.2 units at $M_c^{\text{Lilliefors}}$) despite using the same magnitude types (M_w). Therefore, the M_w estimates are not consistent with each other. Incompleteness may be ruled out as a cause, since the b-value also differs by the same amount in the non-STAI periods. Due to this inconsistency, estimated b-values cannot be compared among these catalogs, for instance to relate the estimated b-value during the sequence using the high-resolution catalog with a reference "background" b-value using HORUS. This finding highlights the need to treat M_w obtained with different conversion relations with utmost care in statistical analysis.

More generally, the choice of the used catalog may influence the analysis because catalog creators often use different approaches to determine earthquake locations and magnitudes.

2.6 Possible contamination by quarry blasts and explosions

An earthquake catalog may be contaminated by unrelated events. Based on recent findings and a discussion that quarry blasts contaminate the MFD and bias the b-value[14,15,16], we obtained locations of quarry blasts in the surrounding of the sequence since May 2012 until August 2021 (see Supplementary Fig. 11). No identified quarry blast or explosion occurred during and within the extracted catalog of the CI2016 sequence. Yet, for good measure, we repeated the analysis shown in Fig. 4 using only night-time seismicity (19:00 to 05:00 UTC, see Supplementary Fig. 13), but cannot find an impact on the b-value trends discussed in the main text.

Supplementary Figures

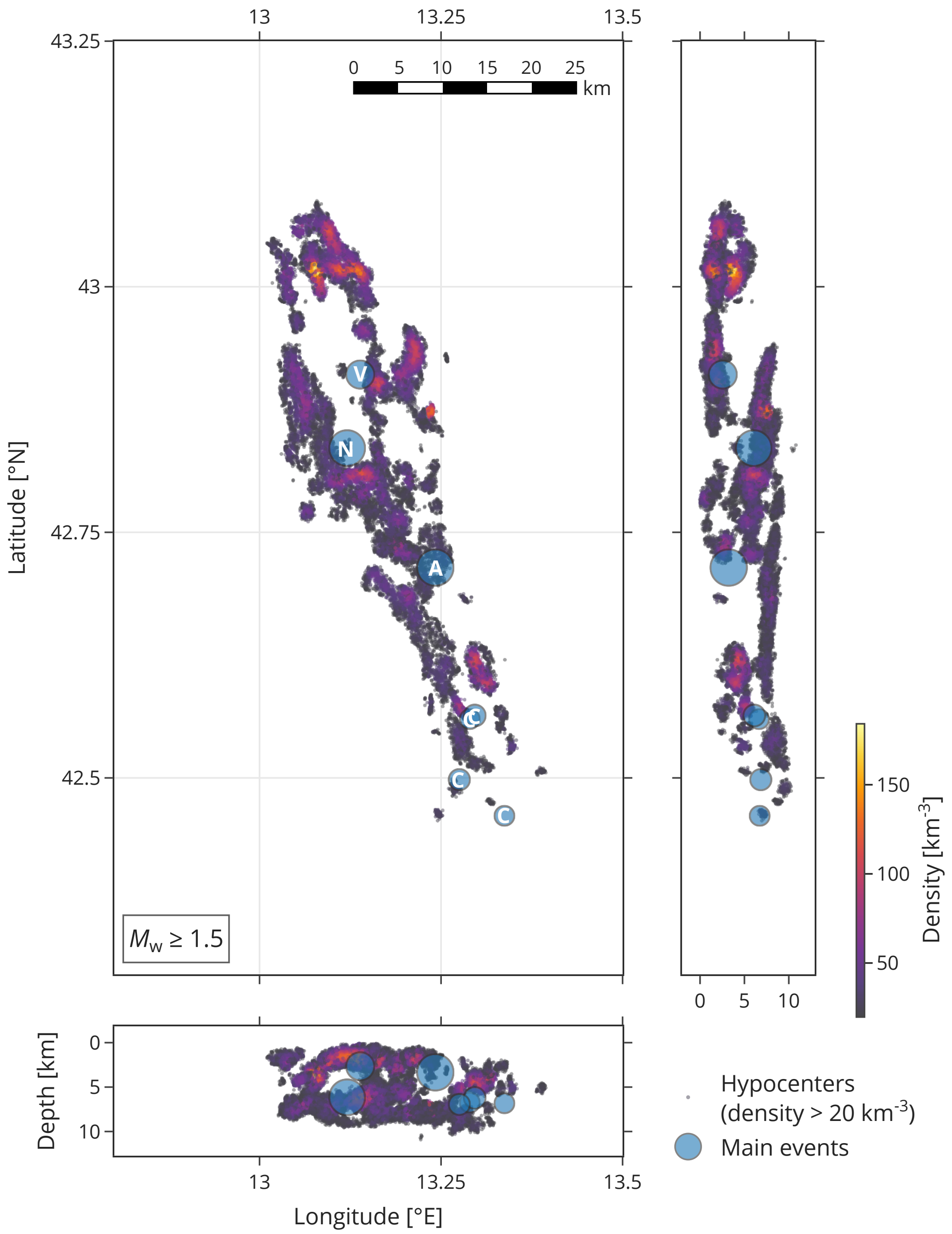

Supplementary Figure 1: Hypocenter density. Map view and depth sections similar to Fig. 1, but for hypocenter density (within a cubic kilometer, km^{-3}; see color bar). The density is shown at every hypocenter whose corresponding density is higher than 20 km^{-3}, which is the median density among all hypocenters. The depth sections are to scale. The main events 'Amatrice', 'Visso', 'Norcia', and four times 'Campotosto' are represented by larger blue circles, annotated with the respective initial letter (A, V, N, C) in the map view.

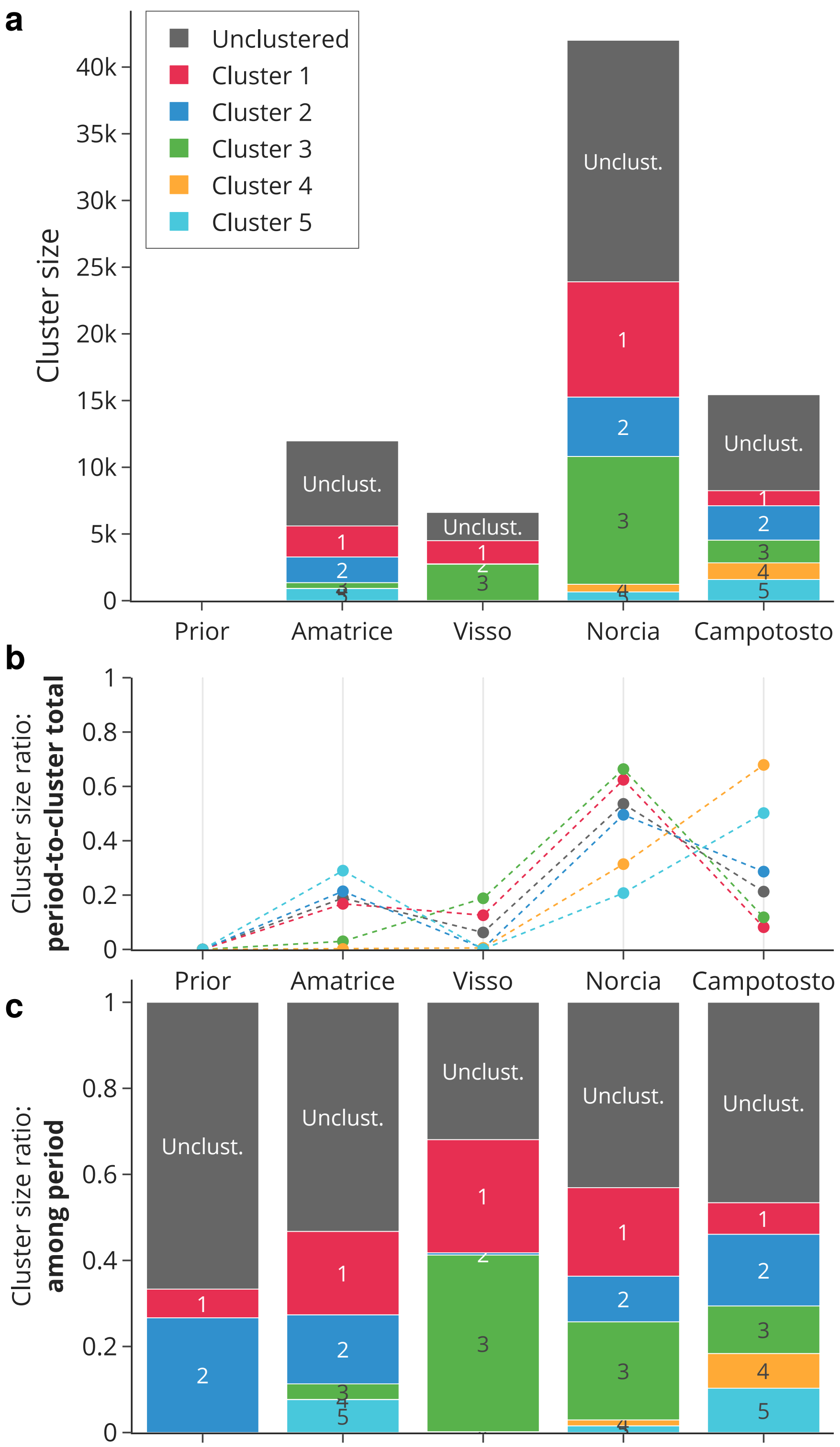

Supplementary Figure 2: Cluster size statistics among the different periods. The periods correspond to the time ranges in Fig. 2 indicated by vertically dashed lines and annotated by the event name. **a** Absolute cluster size; **b** Relative size proportions among periods *per cluster*; **c** Relative size proportions among clusters *per period*. Note that the 'prior' period is only based on 15 earthquakes. See Supplementary Note 1 for more information.

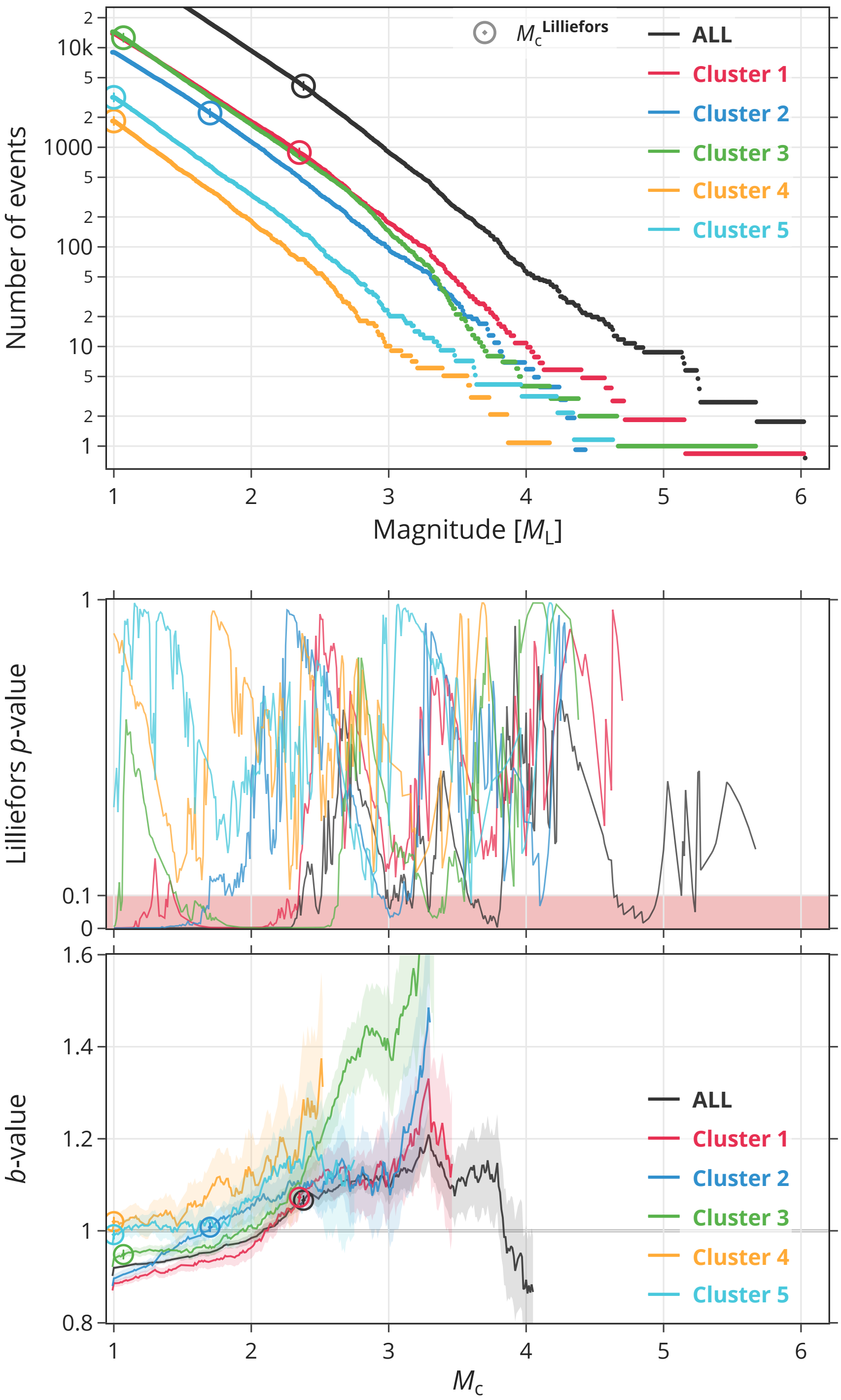

Supplementary Figure 3: Magnitude statistics of individual clusters using M_{L} for all data (black) and entire clusters (see legend). Like Fig. 3, but using local magnitudes, M_{L}. See Supplementary Note 2.3 for a discussion of these results.

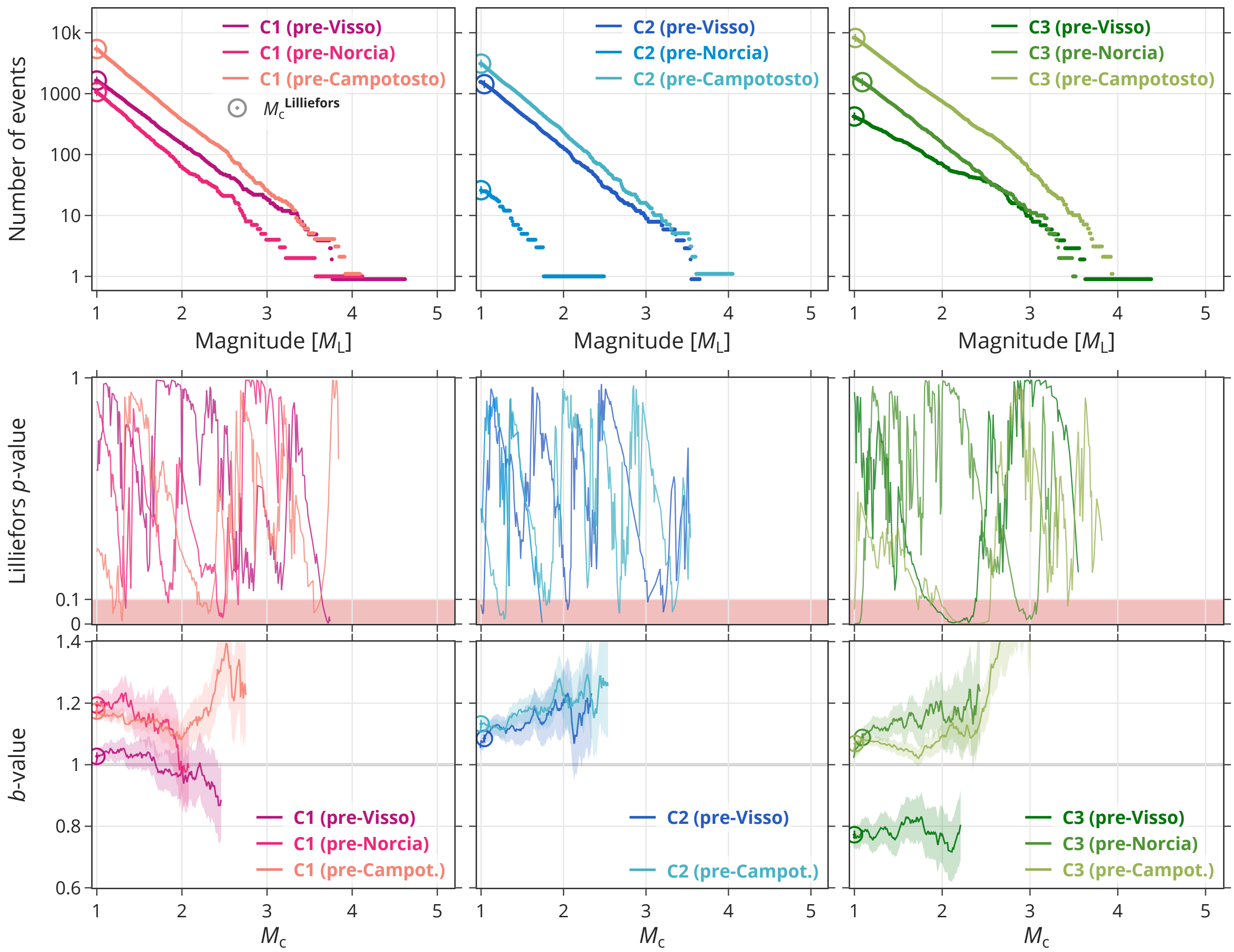

Supplementary Figure 4: Magnitude statistics in three individual periods using M_L. Like Fig. 4, but using local magnitudes, M_L; note that the b-value range in the bottom panels changed compared to Fig. 4.
See Supplementary Note 2.3 for a discussion of these results.

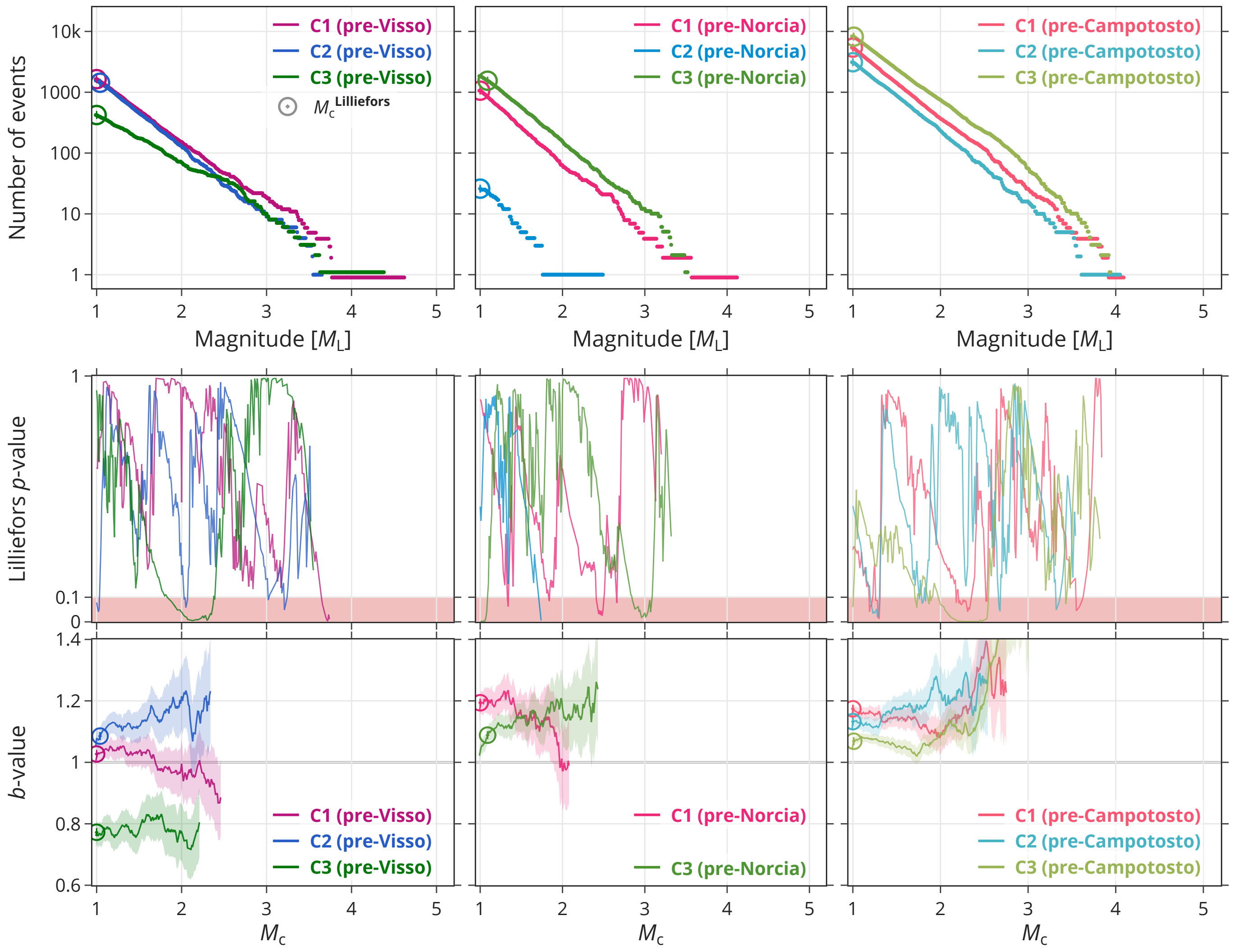

Supplementary Figure 5: Reordering Supplementary Fig. 4 temporally by periods. Like Fig. 5, but using local magnitudes, M_L; note that the b-value range in the bottom panels changed compared to Fig. 4.

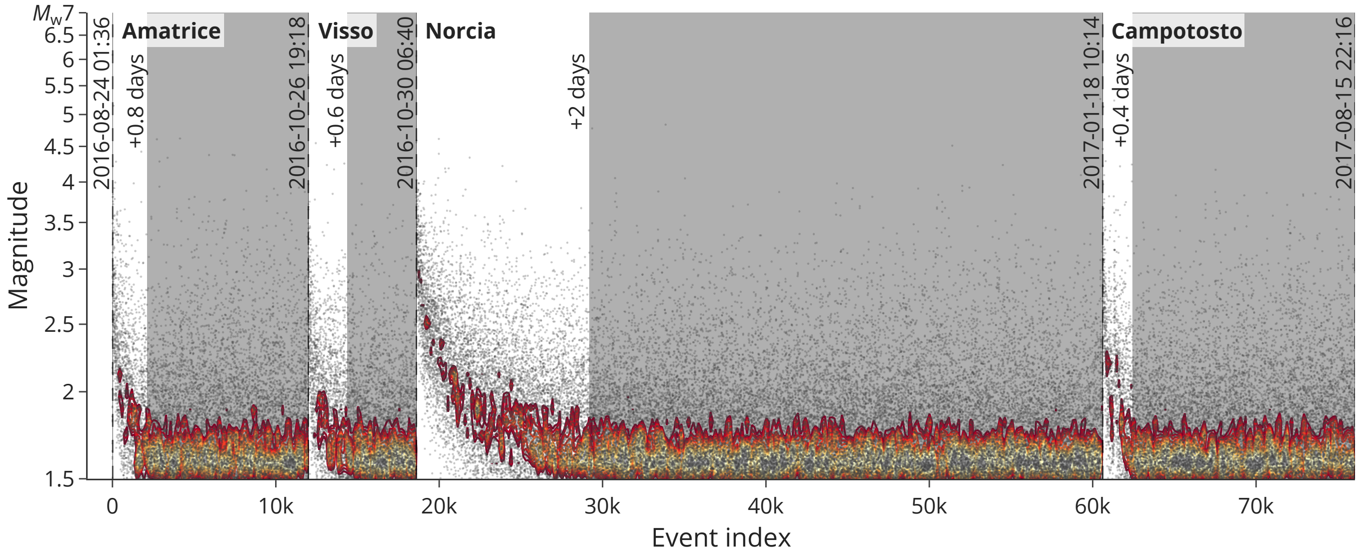

Supplementary Figure 6: Temporal evolution of seismicity highlighting short-term aftershock incompleteness (STAI). The plot is equalized by time (event index) and magnitude (logarithmic axis), as suggested by Agnew [17]. The yellow-red overlay represents a kernel density estimate of the shown points, which reveals the decay of the short-term aftershock incompleteness (STAI) effect to a background level (suggested by W. Ellsworth, pers. comm., 2021). This visualization helps us to define the necessary "safety margin" to reduce the bias due to incompleteness by only using events in the gray-shaded periods (individually for each period).

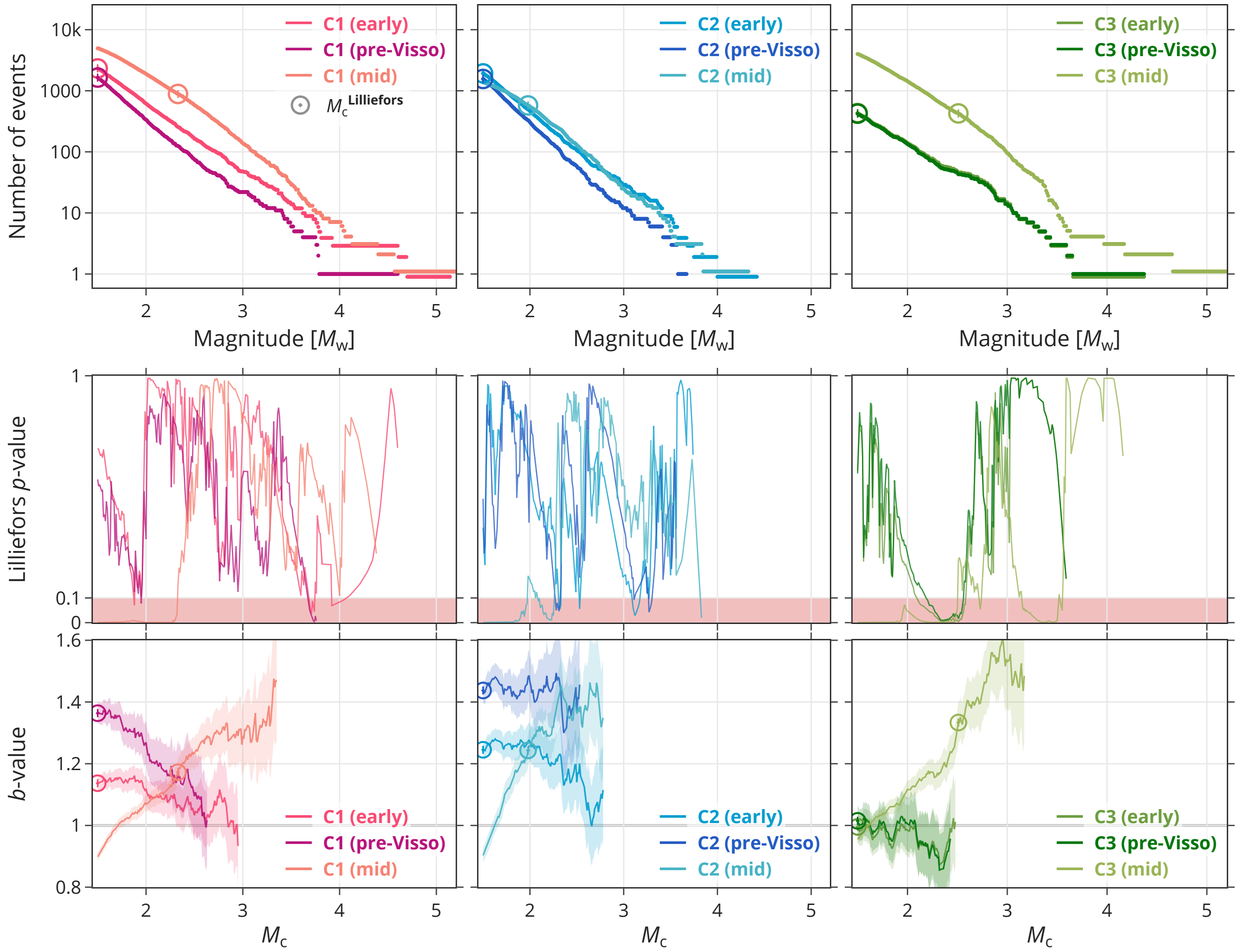

Supplementary Figure 7: Influence of STAI (see Supplementary Fig. 6) on magnitude statistics in the 'early' and 'mid' period.
See Supplementary Note 2.4 for a discussion of these results.

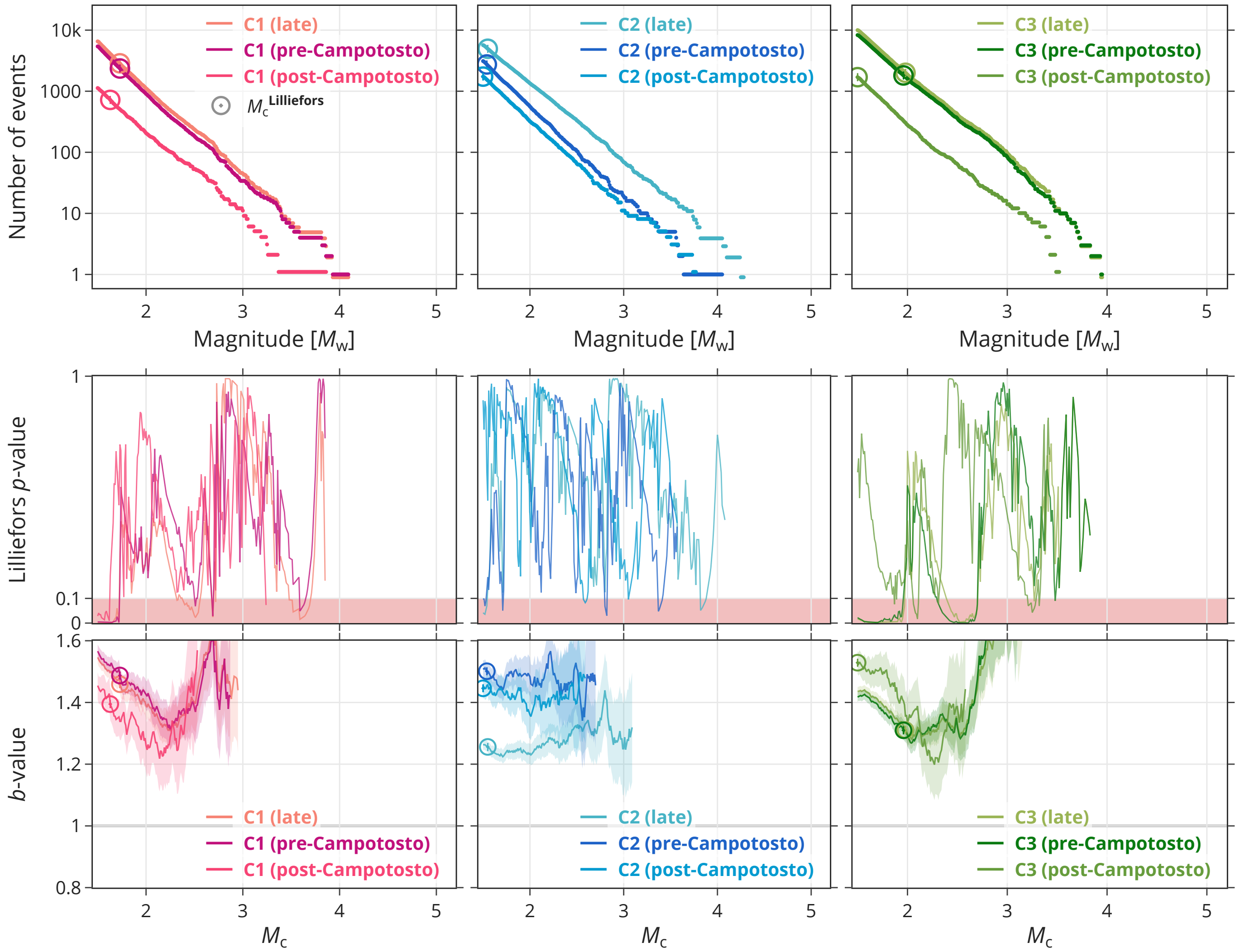

Supplementary Figure 8: Influence of STAI (see Supplementary Fig. 6) on magnitude statistics in the 'late' period. The 'late' period includes the Campotosto subsequence with four M_w5.0–5.5 events.
See Supplementary Note 2.4 for a further discussion of these results.

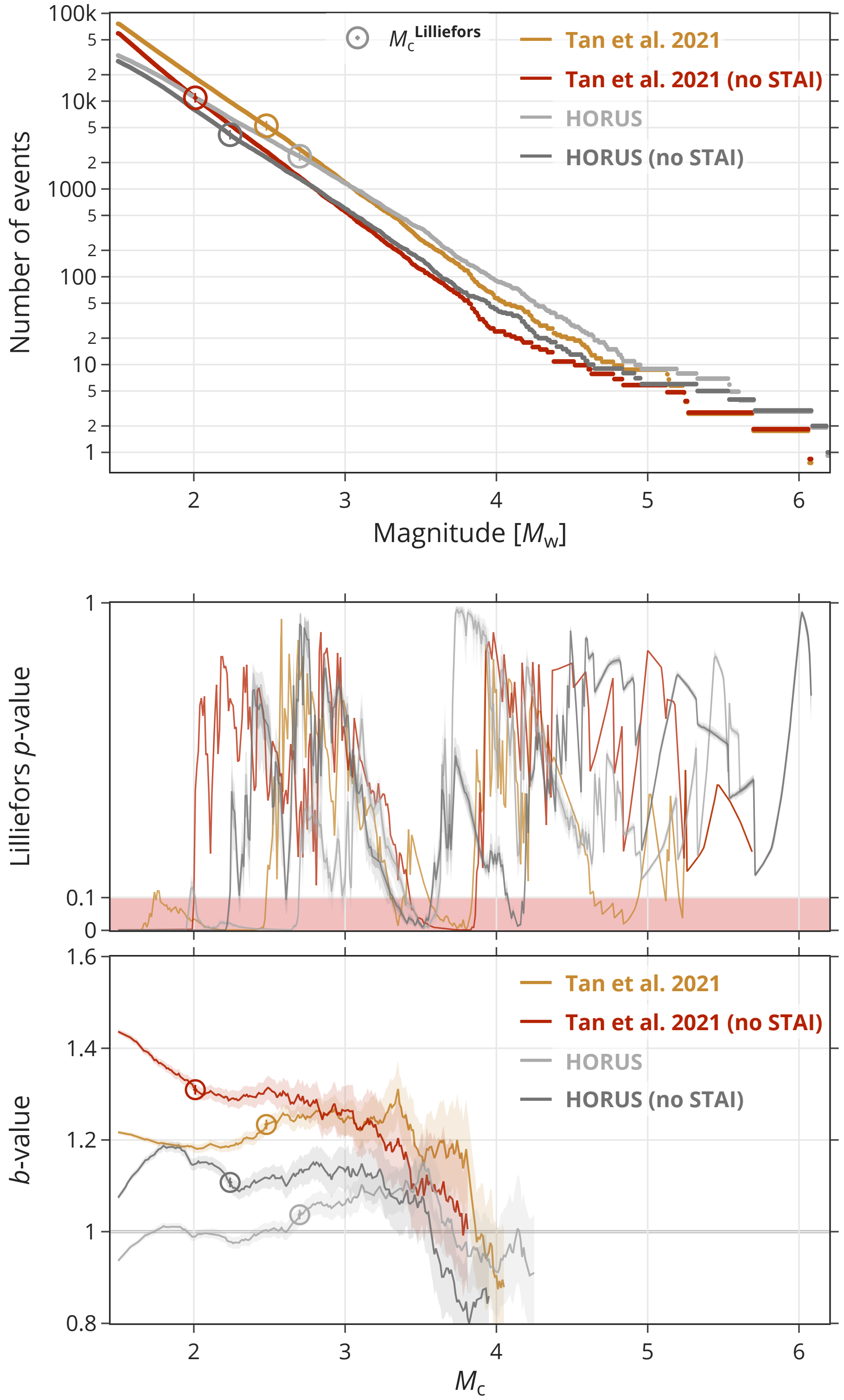

Supplementary Figure 9: Comparison of magnitude statistics for available catalogs of the CI2016 sequence that provide moment magnitudes: Tan et al. [1] and HORUS[8] (`horus.bo.ingv.it`). The performed analysis is analog to Fig. 3 and Supplementary Fig. 3. For both catalogs, the analysis is performed for (i) all events during the sequence, and (ii) events during the sequence without the periods affected by STAI after each main event (i. e., only using the gray-shaded periods in Supplementary Fig. 6), including the main events themselves.
See Supplementary Note 2.5 for more information and a discussion of these results.

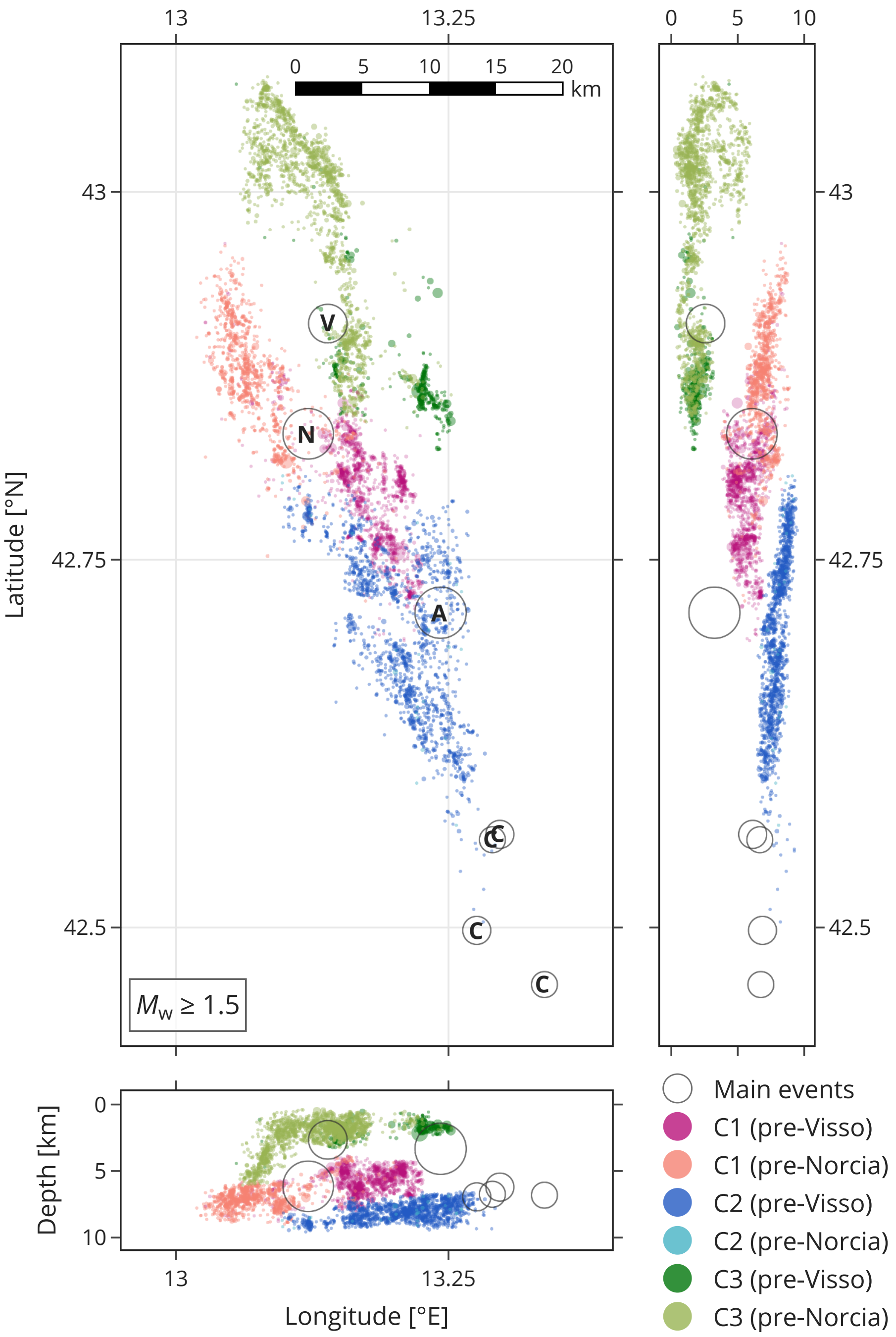

Supplementary Figure 10: Spatial distribution of selected subclusters. Map view and depth sections as in Fig. 1, but showing only the temporal subsets before and after the Visso event ('pre-Visso' and 'pre-Norcia', respectively) for Cluster 1–3 (see legend). Note that the geographical bounds slightly changed compared to Fig. 1 by zooming in. The depth sections are to scale.
The figure shows that (i) Norcia nucleated in between those two subclusters of C1; and (ii) C1's pre-Norcia seismicity occurred mostly at depth, along the north-eastern extension of the subhorizontal shear zone.

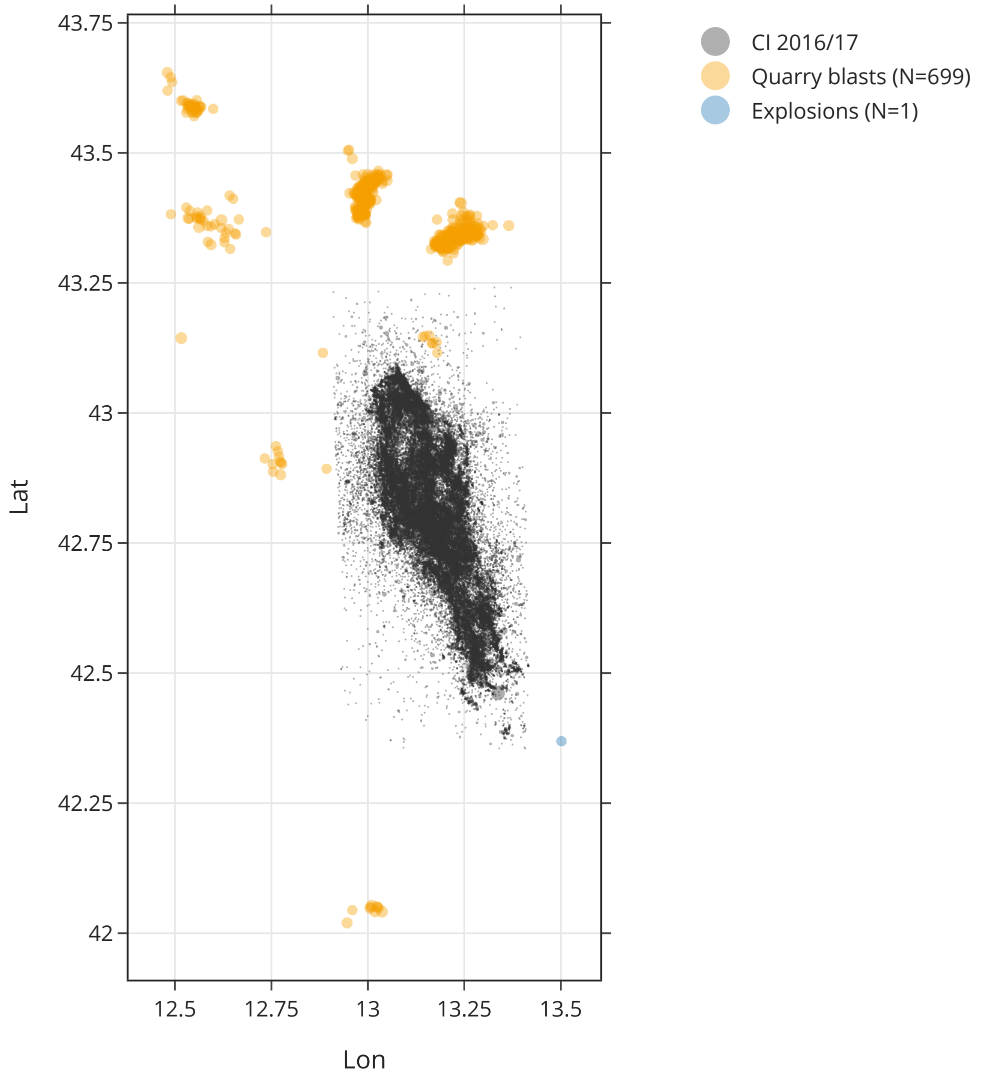

Supplementary Figure 11: Spatial distribution of quarry blasts and CI2016 seismicity. CI2016 seismicity (gray/black) is augmented with locations of quarry blasts and explosions in the region (orange and blue, respectively) since May 2012 until August 2021. None of those occurred during and within the CI2016 sequence.

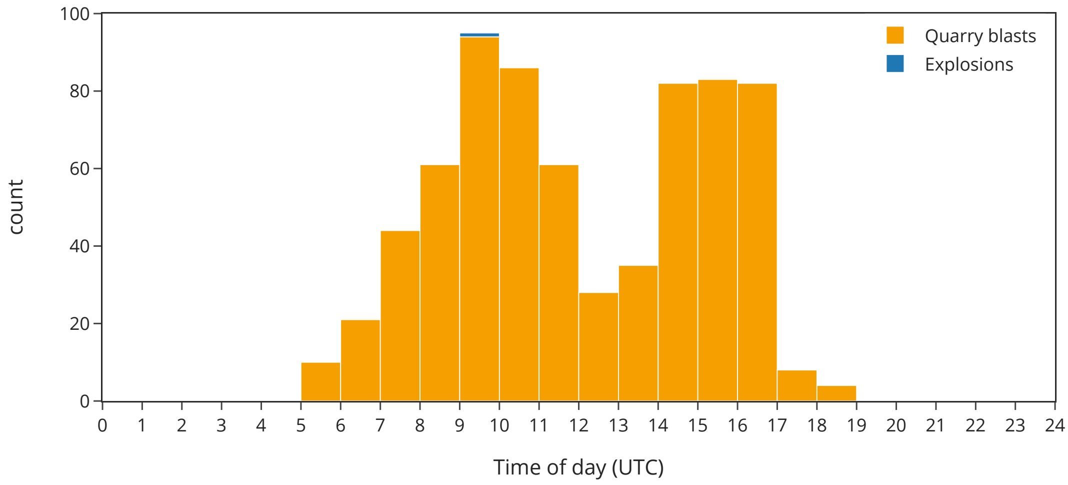

Supplementary Figure 12: Histogram of the diurnal occurrence (UTC Time) of quarry blasts and explosions shown in Supplementary Fig. 11.

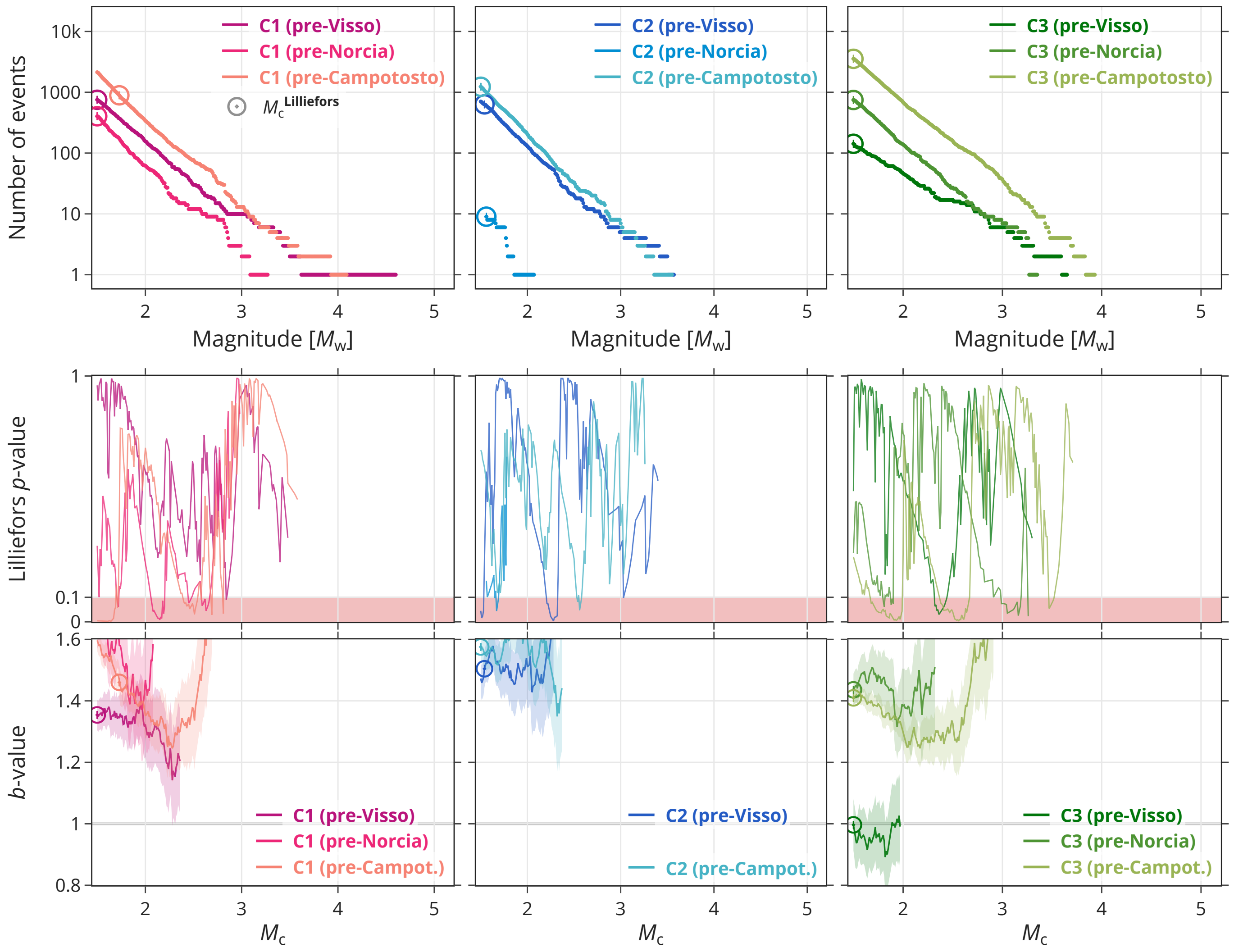

Supplementary Figure 13: Magnitude statistics in individual periods using only nighttime seismicity. Repeating the analysis shown in Fig. 4 (magnitude statistics of the main clusters in individual subperiods) using only nighttime seismicity (19:00 to 05:00 UTC).

Supplementary References

[1] Tan, Y. J. et al. (2021). "Machine-learning-based high-resolution earthquake catalog reveals how complex fault structures were activated during the 2016–2017 central Italy sequence". *The Seismic Record 1*(1), 11–19. DOI: 10.1785/0320210001.

[2] Marzocchi, W. and L. Sandri (2003). "A review and new insights on the estimation of the b-value and its uncertainty". *Annals of Geophysics 46*(6), 1271–1282. DOI: 10.4401/ag-3472.

[3] Marzocchi, W., I. Spassiani, A. Stallone, and M. Taroni (2020). "How to be fooled searching for significant variations of the b-value". *Geophysical Journal International 220*(3), 1845–1856. DOI: 10.1093/gji/ggz541.

[4] Herrmann, M. and W. Marzocchi (2021). "Inconsistencies and lurking pitfalls in the magnitude–frequency distribution of high-resolution earthquake catalogs". *Seismological Research Letters 92*(2A), 909–922. DOI: 10.1785/0220200337.

[5] Munafò, I., L. Malagnini, and L. Chiaraluce (2016). "On the relationship between Mw and ML for small earthquakes". *Bulletin of the Seismological Society of America 106*(5), 2402–2408. DOI: 10.1785/0120160130.

[6] Deichmann, N. (2017). "Theoretical basis for the observed break in ML / Mw scaling between small and large earthquakes". *Bulletin of the Seismological Society of America 107*(2), 505–520. DOI: 10.1785/0120160318.

[7] Grünthal, G., R. Wahlström, and D. Stromeyer (2009). "The unified catalogue of earthquakes in central, northern, and northwestern Europe (CENEC)—updated and expanded to the last millennium". *Journal of Seismology 13*(4), 517–541. DOI: 10.1007/s10950-008-9144-9.

[8] Lolli, B., D. Randazzo, G. Vannucci, and P. Gasperini (2020). "The Homogenized Instrumental Seismic Catalog (HORUS) of Italy from 1960 to present". *Seismological Research Letters 91*(6), 3208–3222. DOI: 10.1785/0220200148.

[9] Gasperini, P., B. Lolli, and G. Vannucci (2013). "Empirical calibration of local magnitude data sets versus moment magnitude in Italy". *Bulletin of the Seismological Society of America 103*(4), 2227–2246. DOI: 10.1785/0120120356.

[10] Malagnini, L. and I. Munafò (2018). "On the relationship between ML and Mw in a broad range: An example from the Apennines, Italy". *Bulletin of the Seismological Society of America 108*(2), 1018–1024. DOI: 10.1785/0120170303.

[11] Hainzl, S. (2016). "Rate-dependent incompleteness of earthquake catalogs". *Seismological Research Letters 87*(2A), 337–344. DOI: 10.1785/0220150211.

[12] Tormann, T., S. Wiemer, S. Metzger, A. J. Michael, and J. L. Hardebeck (2013). "Size distribution of Parkfield's microearthquakes reflects changes in surface creep rate". *Geophysical Journal International 193*(3), 1474–1478. DOI: 10.1093/gji/ggt093.

[13] Van der Elst, N. J. (2021). "b-positive : A robust estimator of aftershock magnitude distribution in transiently incomplete catalogs". *Journal of Geophysical Research: Solid Earth 126*(2), 1–19. DOI: 10.1029/2020JB021027.

[14] Gulia, L. and P. Gasperini (2021). "Contamination of frequency–magnitude slope (b-value) by quarry blasts: An example for Italy". *Seismological Research Letters 92*(6), 3538–3551. DOI: 10.1785/0220210080.

[15] Taroni, M., J. Zhuang, and W. Marzocchi (2022). "Reply to "Comment on 'High-definition mapping of the Gutenberg–Richter b-value and its relevance: A case study in Italy' by M. Taroni, J. Zhuang, and W. Marzocchi" by Laura Gulia, Paolo Gasperini, and Stefan Wiemer". *Seismological Research Letters 93*(2A), 1095–1097. DOI: 10.1785/0220210244.

[16] Gulia, L., P. Gasperini, and S. Wiemer (2022). “Comment on “High-definition mapping of the Gutenberg–Richter b-value and its relevance: A case study in Italy” by M. Taroni, J. Zhuang, and W. Marzocchi”. *Seismological Research Letters 93*(2A), 1089–1094. DOI: 10.1785/0220210159.
[17] Agnew, D. C. (2015). “Equalized plot scales for exploring seismicity data”. *Seismological Research Letters 86*(5), 1412–1423. DOI: 10.1785/0220150054.
