# Supplementary material for: Revealing the spatiotemporal complexity of the magnitude distribution and b-value during an earthquake sequence
Source: Nat Commun. 2022 Aug 29;13:5087. doi: 10.1038/s41467-022-32755-6 (PMC9424211; doi:10.1038/s41467-022-32755-6)
Supplement: Supplementary file 3 — Description of Additional Supplementary Files [file 41467_2022_32755_MOESM3_ESM.pdf]

Description of Additional Supplementary Files

File Name: Supplementary Data 1
Description: Extracted events from the earthquake catalogue of Tan et al. 2021 with associated cluster IDs obtained in this study.
